# Retinal glial responses to optic nerve crush are attenuated in *Bax*-deficient mice and modulated by purinergic signaling pathways

**DOI:** 10.1186/s12974-016-0558-y

**Published:** 2016-04-28

**Authors:** Caitlin E. Mac Nair, Cassandra L. Schlamp, Angela D. Montgomery, Valery I. Shestopalov, Robert W. Nickells

**Affiliations:** Department of Ophthalmology and Visual Sciences, University of Wisconsin, 571A Medical Sciences—1300 University Ave, Madison, WI 53706 USA; Cellular and Molecular Pathology Graduate Program, University of Wisconsin—Madison, 3170-10K/L MFCB, 1685 Highland Avenue, Madison, WI 53705 USA; Department of Ophthalmology, University of Miami Miller School of Medicine, 900 N.W. 17th Street, Miami, FL 33136 USA; Department of Cell Biology and Anatomy, University of Miami Miller School of Medicine, 900 N.W. 17th Street, Miami, FL 33136 USA

**Keywords:** Retinal ganglion cell, Optic nerve damage, Microglia, Macroglia, P2X Receptor, PANX1, BAX, Neuroinflammation

## Abstract

**Background:**

Retinal ganglion cell (RGC) soma death is a consequence of optic nerve damage, including in optic neuropathies like glaucoma. The activation of the innate immune network in the retina after nerve damage has been linked to RGC pathology. Since the eye is immune privileged, innate immune functions are the responsibility of the glia, specifically the microglia, astrocytes, and Müller cells that populate the retina. Glial activation, leading to the production of inflammatory cytokines, is a hallmark feature of retinal injury resulting from optic nerve damage and purported to elicit secondary degeneration of RGC somas.

**Methods:**

A mouse model of optic nerve crush (ONC) was used to study retinal glial activation responses. RGC apoptosis was blocked using *Bax*-deficient mice. Glial activation responses were monitored by quantitative PCR and immunofluorescent labeling in retinal sections of activation markers. ATP signaling pathways were interrogated using P2X receptor agonists and antagonists and *Pannexin 1* (*Panx1*)-deficient mice with RGC-specific deletion.

**Results:**

ONC induced activation of both macroglia and microglia in the retina, and both these responses were dramatically muted if RGC death was blocked by deletion of the *Bax* gene. Macroglial, but not microglial, activation was modulated by purinergic receptor activation. Release of ATP after optic nerve damage was not mediated by PANX1 channels in RGCs.

**Conclusions:**

RGC death in response to ONC plays a principal stimulatory role in the retinal glial activation response.

**Electronic supplementary material:**

The online version of this article (doi:10.1186/s12974-016-0558-y) contains supplementary material, which is available to authorized users.

## Background

Glaucoma is a group of optic neuropathies characterized by the degeneration of the optic nerve and loss of retinal ganglion cells (RGCs) [1–3]. Among the mechanisms that have been correlated with glaucomatous neurodegeneration is the activation of the retinal innate immune response [4–6], which has been replicated in animal models of glaucoma and following optic nerve trauma [7–11]. Since the eye is immune privileged and protected from invading peripheral immune cells [12], it relies on the adaptation of the retinal glial cells to incorporate innate immune functions in response to injury. In the retina, there are three types of glial cells that maintain health and function. Microglia are found in the plexiform layers where neurons form synapses [8], and astrocytes contact the unmyelinated axons of RGCs as they pass over the retina in the nerve fiber layer [13]. The third type is the Müller glia, which are the most abundant glial population in the eye [14–16]. Müller glia span the full thickness of the retina and form connections with every cell type involved in processing light signals [14, 17].

The retinal glia undergo distinct morphological and behavioral changes to enter an activated state in response to injuries such as optic nerve crush, optic nerve transection, and in animal models of glaucoma [8, 13, 18–20]. Microglia become “amoeboid” and exhibit proliferative potential, retracted processes with enlarged somas [13, 21], and they upregulate factors including allograft inflammatory factor 1 (*Aif1/Iba1*) and *Cd68* [22, 23]. Müller cells and astrocytes, while non-proliferative, upregulate glial fibrillary acidic protein (*Gfap*) and nestin (*Nes*) [24–27], and become hypertrophic [28]. Activated glia have been shown to phagocytose cellular debris, generate cytokines, and present antigens [29–34].

Despite the well-documented activation response of the retinal glia following axonal injury, the consequence of this activation response on RGC survival continues to be debated. One model has been that of “secondary degeneration”, which predicts that RGC neurodegeneration occurs in two waves. First, axonal injury causes intrinsic apoptosis in a subset of critically damaged RGCs, after which a second wave of extrinsic apoptotic death is triggered by inflammatory molecules produced by activated retinal glia. The phenomenon of secondary degeneration was initially developed following a series of studies utilizing partial optic nerve crush, or partial optic nerve axotomy. In this damage paradigm, RGCs with presumably intact axons also exhibit degeneration [9, 35–41]. The supposition that activated glial cells mediate the process of secondary degeneration was supported by improved RGC survival after acute optic nerve injury when glial activation was attenuated by the anti-inflammatory action of minocycline [42, 43], and when the signaling potential of tumor necrosis factor alpha (TNFα) through TNFα receptor 1 (TNFR1) was blocked [44]. In chronic optic nerve damage conditions, such as glaucoma, the phenomenon of secondary degeneration is less apparent; however, RGC survival in experimental glaucoma was also improved by treatment with minocycline [20, 43, 45] and with the TNFα decoy receptor, Etanercept [46], or in mice lacking the *Tnf* gene [47].

Additionally, in studies evaluating changes of the retinal transcriptome in both acute and chronic models of optic nerve damage, the involvement of neuroinflammatory pathways are almost universally identified [48, 49]. Interestingly, RGC death following optic nerve injury and cytokine-mediated damage occurs by distinct mechanisms: the former occurs through intrinsic apoptosis and is mediated by BAX [50, 51], while the latter occurs through extrinsic apoptotic pathways that are predicted to be BAX independent. Paradoxically, RGC death is completely abrogated in *Bax*^−/−^ mice after both acute and chronic optic nerve damage [50–54], a pattern that would be inconsistent with secondary activation of degeneration mediated through cytokine activation of extrinsic apoptosis. This might suggest that *Bax*^−/−^ mice are resistant to cytokine-mediated damage; however, like wild types, *Bax*^−/−^ mice retain susceptibility to TNFα [55], indicating that the extrinsic apoptotic pathways remain functional. It is therefore possible that the glial cells in *Bax*^−/−^ mice exhibit an altered activation state in which the damaging cytokines that initiate extrinsic apoptosis are not produced. To further examine this phenomenon in the *Bax*^−/−^ mice, we explored the mechanisms of glial activation using the mouse model of optic nerve crush (ONC).

In this model, the site of damage in the optic nerve is “distant” from the retina, suggesting a secondary signal within the retina is required to activate the resident glia. In the context of secondary degeneration, the likely source of activating signals is the RGC somas that are lost during the primary wave of degeneration. A variety of signaling molecules have been attributed to dying neurons, including (but not limited to) cytokines, reactive oxygen species, damage-associated molecular patterns (DAMPs), excessive neurotransmitters, and heat shock proteins [56–59]. An attractive candidate in the retina, however, is ATP. There is growing evidence that this nucleotide is released by dying neurons and signals extracellularly as a danger signal [60–62]. Although not studied in models of acute optic nerve damage, there is evidence that cells affected in more moderate models of intraocular pressure-mediated stress exhibit purinergic signaling responses. Elevated levels of ATP have been found in the aqueous humor of glaucoma patients [63], the vitreal compartment of bovine retinal eye cups subjected to increased hydrostatic pressure [64], and following pressure-induced mechanical deformation of neuronal cultures [65]. The release of ATP can also be blocked with probenecid, carbenoxolone, and ^10^panx, implicating a role for pannexin 1 (PANX1) hemichannels in mediating ATP release [65–67].

Once ATP reaches the extracellular space, this nucleotide can signal through two families of receptors, called P1 and P2. While P1 receptors are only activated by adenosine, P2 receptors respond to extracellular nucleotides including ATP [68]. P2 receptors are further categorized as metabotropic G-protein-coupled P2Y receptors and ionotropic P2X receptors [69]. A number of the P2X receptor subunits are expressed in the retina [68–70], but a considerable amount of research has been conducted on the P2X7 receptor (P2X7R). Stimulating P2X7R has been linked to the activation of both microglia and macroglia [71, 72], as well as the production of inflammatory cytokines [73], while inhibiting the receptor has reduced macrophage recruitment and cytokine production at the site of optic nerve crush in rats [74]. The activation of P2X7R in vitro and in vivo has also been shown to damage RGCs, although the glial contribution to RGC damage was not examined in vivo, leaving open the possibility that glial activation (initiated by ATP) causes secondary degeneration of RGCs [75–77].

In this study, we examined how blocking RGC death in the crush model altered the activation state of microglia and macroglia, as a function of activation markers at the mRNA and protein levels. We found that glial activation was attenuated when cell death was blocked in *Bax*^−/−^ mice. Additionally, an ATP agonist elicited macroglial activation without optic nerve trauma, while an ATP antagonist for purinergic receptors attenuated the activation response after crush.

## Methods

### Animals

Adult mice (Jackson Laboratory, Bar Harbor, ME) were handled in accordance with the Association for Research in Vision and Ophthalmology statement on the use of animals in research. All experimental protocols and the ethical care of the mice were reviewed and approved by the Institutional Animal Care and Use Committee of the University of Wisconsin. Mice were housed in microisolator cages and kept on a 12-h light/dark cycle and maintained on a 4 % fat diet (8604 M/R; Harland Teklad, Madison, WI). *Bax-*deficient mice were generated from breeding *Bax*^*+/−*^ animals on a C57BL/6J background. Mice harboring LoxP sites flanking exons 3 and 4 in the *Panx1* gene (*Panx1*^*fl/fl*^), as previously described [78], were used to generate *Panx1* total knockout mice and RGC conditional knockouts. For complete knockout mice, *Panx1*^*fl/fl*^ animals were crossed with transgenic mice carrying CRE recombinase under the control of the CMV immediate early promoter. For RGC-selective deletion of *Panx1*, *Panx1*^*fl/fl*^ mice received an intraocular injection of a replication-deficient AAV2 virus carrying a CRE expression cassette (AAV2-Cre/GFP, Vector Biolabs, Philadelphia, PA and University of North Carolina Viral Vector Core, Chapel Hill, NC), which transduces approximately 85 % of the RGCs with only minimal transduction of some Müller cells [79, 80]. All genotypes were on the C57BL/6 background.

### Optic nerve crush surgery and intraocular injections

ONC was performed as previously described [55, 81]. Briefly, mice were anesthetized with ketamine (120 mg/kg) and xylazine (11.3 mg/kg), and the eye was numbed with a drop of 0.5 % proparacaine hydrochloride (Akorn, Lake Forest, IL). A lateral canthotomy was performed followed by an incision through the conjunctiva at the limbal junction, and the optic nerve was exposed and clamped for 3 s using self-closing N7 forceps (Fine Science Tools, Foster City, CA). After surgery, the eye was covered with triple antibiotic ointment, and a subcutaneous injection of Buprenex (0.2 mg/kg) was delivered to alleviate pain. The right eye was left as an untreated control for each experiment.

Intraocular injections were performed as previously described [55, 81]. Briefly, mice were anesthetized with ketamine/xylazine, and a drop of proparacaine was applied to numb the eye. A small hole was made through the conjunctiva and scleral tissue with a 30G needle, and then a 35G beveled Nanofil needle attached to a Nanofil syringe (World Precision Instruments, Inc, Sarasota, FL) was inserted through the hole and a 1-μl volume of PBS, 40 mM *N*-*methyl*-d-asparate (NMDA), 250 μM 2′(3′)-O-(4-benzoyl)benzoyl adenosine 5′-triphosphate triethylammonium salt (BzATP; Sigma, St. Louis, MO), 250 μM oxidized ATP (oxATP; Sigma), or AAV2-Cre/GFP was slowly delivered into the vitreous over 30 s. Care was taken not to damage the lens. After delivery, the needle was held in the eye for an additional 30 s before being retracted. After surgery, the eye was covered with triple antibiotic ointment, a subcutaneous injection of Buprenex was delivered to alleviate pain, and the mouse was allowed to recover. The virus was diluted 1:10 in PBS containing 5 % glycerol prior to injection, and mice were housed for a minimum of 4 weeks after gene delivery to allow for expression of the transgene.

### RNA isolation and quantitative analysis of mRNA expression by quantitative PCR

RNA isolations were performed as previously described [55]. Briefly, mice were euthanized with a lethal overdose of pentobarbital sodium, and retinal tissue was collected and flash frozen on dry ice. Total RNA was isolated from a minimum of three pooled retinas, and 4 μg were treated with DNase I (Promega, Madison, WI), purified by phenol/chloroform extraction, and converted to (complementary DNA) cDNA with oligo(dT) 15 primers and Moloney murine leukemia virus (M-MLV) reverse transcriptase (Promega). The cDNA samples were then diluted and an equivalent amount of 2 ng of mRNA was analyzed by quantitative PCR (QPCR) for changes in gene expression of *Aif1*, *Cd68*, *Gfap, Nes*, *Nrn1*, *Panx1*, and *Sncg. S16* ribosomal protein mRNA was used as a reference gene. The primer sequences are listed in Table 1, and the identity of all products was confirmed by sequence analysis. The cDNA was added to diluted SYBR Green PCR master mix (Applied Biosystems, Grand Island, NY) with 0.25 μM of each primer in a 20 μl reaction volume. Cycling conditions were 95 °C (15 s) and 60 °C (60 s) for 40 cycles with a dissociation step. Each cDNA sample was run in triplicate on an ABI 7300 Real-Time PCR system (Applied Biosystems), and absolute transcript abundance was determined using a standard curve of the target molecule run on the same array. Data from different samples were normalized to *S16*.

**Table 1:** Quantitative PCR primer sequences

Gene Name	Primer sequence 5′ ➔ 3′	Size (bp)
*Aif1*	F: AGAGAGGTGTCCAGTGGC R: CCCCACCGTGTGACCTCC	200
*Cd68*	F: GGACTACATGGCGGTGGAAT R: GCAAGAGAAACATGGCCCGA	229
*Gfap*	F: CAAACTGGCTGATGTCTACC R: AGAACTGGATCTCCTCATCC	269
*Nes*	F: GGGCCAGCACTCTTAGCTTTGATA R: TGAGCCTTCAGGGTGATCCAG	106
*Nrn1*	F: TTCACTGATCCTCGCGGTGC R: TACTTTCGCCCCTTCCTGGC	238
*Panx1*	F: GCTGCACAAGTTCTTCCCCT R: ATCTCGAGCACTCTTGGCAG	175
*Sncg*	F: GACCAAGCAGGGAGTAACGG R: TCCAAGTCCTCCTTGCGCAC	240
*S16*	F: CACTGCAAACGGGGAAATGG R: TGAGATGGACTGTCGGATGG	198

### Immunofluorescence and microscopy

Immunofluorescence was performed on 10-μm thick frozen sections as previously described [55]. Slides were rinsed in PBS and then blocked in 0.1 % Triton-X and 2 % BSA in PBS for 1 h at room temperature. Primary antibodies for GFAP (Dako, Cat# Z0334) or AIF1 (Wako, Cat# 019-19741) were incubated at 1:1000 either overnight (GFAP) or for 3 days (AIF1). After washing with PBS, secondary antibodies conjugated to Texas Red or FITC (Jackson ImmunoResearch Inc., West Grove, PA) were diluted 1:1000 and incubated in the dark for 2 h at room temperature. Slides were thoroughly rinsed in PBS before being incubated with 300 ng/ml DAPI for 5 min at room temperature. Finally, the slides were rinsed in PBS and coverslipped with Immu-Mount (Fisher Scientific, Waltham, MA) and stored at 4 °C in the dark. Whole mounts labeled with AIF1 were processed in the same manner, with the exception that retinas were mounted on Superfrost Plus Slides (Fisher Scientific) prior to staining. Images were taken at ×200 and ×400 magnification from the mid-peripheral retina. Microglial counts were obtained from at least six fields per retina of approximately 0.04 mm^2^ in size. For Sholl analyses of retinal microglia in the ganglion cell layer, a grid of concentric circles of 20, 40, 60, and 80 μm in diameter was laid over the cell nucleus of AIF1-staining cells in images from retinal whole mounts and the numbers of intersections of processes was counted. A minimum of 50 cells was measured for each condition. Sizes of microglia in non-damaged retinas of both genotypes of mice were measured from images using ImageJ (v1.45s), while the cells were scored as either amoeboid-like or ramified-like. A minimum of 106 cells were measured and scored for each genotype. All immunofluorescent photographs were acquired using a Zeiss Axioplan 2 Imaging microscope (Carl Zeiss Microimaging, Inc., Thornwood, NY) with a digital black and white camera. Images were analyzed using the Zeiss Axiovision Image Analysis software v4.6 (Carl Zeiss Microimaging, Inc.).

### Statistical analyses

Means from QPCR quantification are reported with the standard deviation of the mean. Microglial counts and Sholl analyses are reported with standard error of the mean. Statistical significance between two means was determined using a two-sided Student’s *t* test, ANOVA was used to compare means from multiple samples, and a chi-squared test was used to evaluate the distribution of microglial morphology in control retinas. *P* values were considered significant at a value equal to or less than 0.05.

## Results

### Microglial activation is attenuated in *Bax*^−/−^ mice after crush

**Fig. 1:**
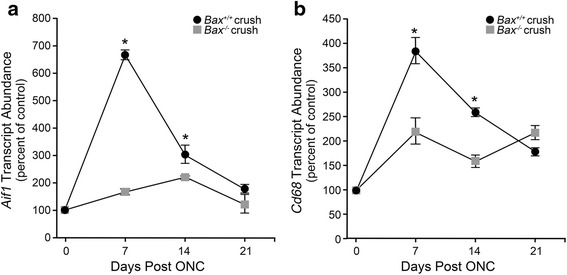
Expression of *Aif1* and *Cd68* are attenuated in *Bax*-deficient mice after optic nerve crush. Wild-type and *Bax* ^−/−^ mice were subjected to crush injury and evaluated for microglial activation markers. **a** In wild-type mice, the mRNA expression of *Aif1* peaked by 7 days post-injury, and then declined at 14 days and again at 21 days. In the *Bax* ^−/−^ mice, *Aif1* expression was significantly attenuated relative to wild-type mice at 7 and 14 days. By 21 days, the levels of *Aif1* appeared to be returning to baseline in both genotypes, and were no longer significantly different (*P* = 0.07). **b** The change in *Cd68* expression followed a similar trend as *Aif1*, with levels peaking in the wild-type mice at 7 days before declining at 14 and again at 21 days. The *Bax* ^−/−^ mice, however, exhibited a significant attenuation in *Cd68* expression at 7 and 14 days post-crush. By 21 days, expression in the knockout mice was significantly elevated over the wild types; however, expression in both genotypes remained low. Data is presented as mean ± SD. *P < 0.005. For each genotype at each time point, *n* ≥ 3

The central hypothesis of secondary degeneration is that activation of the retinal neuroinflammatory response requires an initial wave of RGC somatic degeneration principally affected by axonal damage in the optic nerve. To test this hypothesis, we used *Bax*-deficient mice, which exhibit complete resistance to RGC soma death in an acute optic nerve crush paradigm. We first evaluated the *Bax*^−/−^ mice for expression of the microglial activation marker, *Aif1*, and the microglial/macrophage marker *Cd68* [23]. By 7 days post-injury, *Aif1* expression in wild-type mice was dramatically elevated in the crushed eye over the contralateral eye, while *Bax*-deficient mice only showed a modest increase in *Aif1* expression (Fig. 1a, *P* < 0.0001 for *Bax*^−/−^ mice compared to wild types). By 14 days, *Aif1* expression declined in wild-type mice but remained significantly higher than the mRNA levels in *Bax*-deficient retinas, and at 21 days, microglial activation trended towards baseline levels in both genotypes and was no longer statistically significant. The expression of *Cd68* followed a similar trend as *Aif1*, with levels rising at 7 days post-crush to a significantly higher level in the wild-type mice relative to the *Bax*^−/−^ mice (Fig. 1b, *P* < 0.005). By 14 days, expression began to decline in the crushed eye of both genotypes, although mRNA levels remained significantly higher in the injured retinas of wild-type mice. At 21 days, *Cd68* expression in the knockout mice was slightly higher than the wild types, although the mRNA abundance was relatively low in both genotypes.

**Fig. 2:**
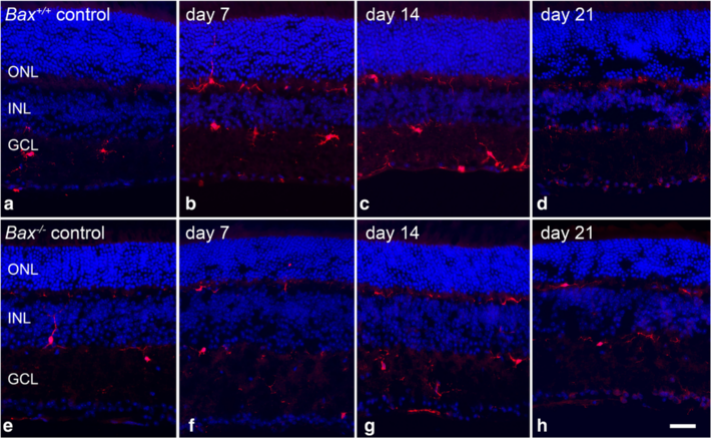
Upregulation of AIF1 is attenuated in *Bax*-deficient mice after optic nerve crush. Cross sections of eye cups were analyzed for changes in AIF1 expression after crush injury. **a**–**d** Although a baseline level of AIF1-positive cells (*red label*) populated the retina in control eyes, following optic nerve crush *Bax* ^+/+^ retinas exhibited an increase in the number of AIF1-positive cells. Microglial cells appeared in the inner plexiform layer (IPL), outer plexiform layer (IPL), and ganglion cell layer (GCL). **e**–**h** In *Bax* ^−/−^ mice, the amount of AIF1 labeling was significantly attenuated after crush, and the intensity of AIF1 labeling did not appear to change. Inner nuclear layer (INL), outer nuclear layer (ONL). DAPI nuclear counterstain (*blue label*). *Scale bar* 50 μm. For each genotype at each time point, *n* ≥ 3

**Fig. 3:**
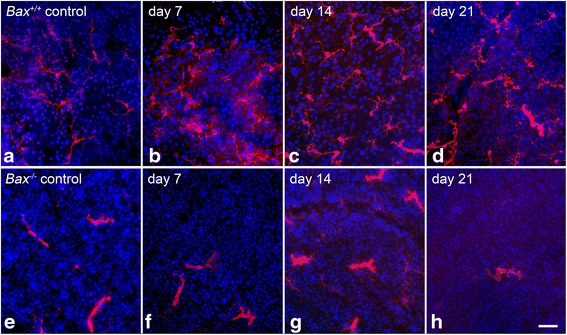
Immunofluorescent images of whole-mounted retinas stained for AIF1. Representative nerve fiber layer images of retinas from wild-type and *Bax* ^−/−^ mice stained for AIF1 (*red label*) at 7, 14, and 21 days post-crush. These are representative of the images used to quantify the number of microglia present after crush. **a**–**d** In wild-type mice, a baseline resting microglial population was present in the retina. These microglia exhibited small somas with numerous long processes. After crush, the number of microglia increased, and the morphology transitioned to an amoeboid state as the cell somas thickened and the processes retracted. By 21 days, while there was still a prominent population of AIF1-positive microglia, the number of cells was beginning to decline. **e**–**h** The number and morphology of microglia in the *Bax* ^−/−^ mice contrasted starkly with the population in the wild types. The number of microglia in the knockout mice did not increase by 7 days after crush, and after a modest increase in AIF1 labeling at 14 days, the number of microglia dropped considerably. Interestingly, the morphology of microglia in the *Bax* ^−/−^ mice appeared amoeboid, even in the control eyes, characteristic of an early activation state. These microglia exhibited very few processes and thickened somas. Microglial counts were obtained from at least six images, and each image is approximately 0.04 mm^2^. DAPI nuclear counterstain (*blue label*). *Scale bar* 50 μm. For each genotype at each time point, *n* ≥ 3

**Fig. 4:**
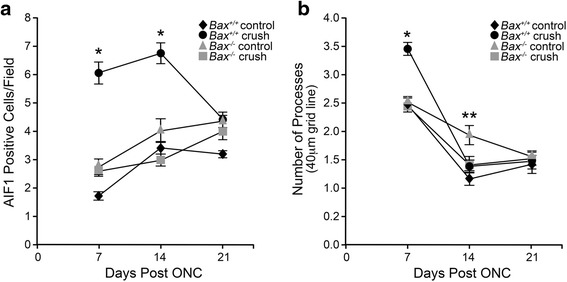
Microglial changes in the peripheral retinal nerve fiber layer of *Bax* ^−/−^ mice after crush. **a** The number of AIF1-positive cells were quantified from retinal whole mounts of wild-type and *Bax* ^−/−^ mice after crush. Consistent with past literature, the number of microglia in the wild-type retinas rose dramatically by 7 days after crush, and remained significantly elevated at 14 days before declining at 21 days. Microglial counts in the *Bax* ^−/−^ mice were significantly attenuated relative to the wild types, and although a modest increase was quantified in both the experimental and control eyes (suggesting a contralateral effect from crush injury), the number of microglia in the crushed retinas of *Bax* ^−/−^ mice was significantly attenuated at 7 and 14 days post-crush. By 21 days, the microglial counts were no longer statistically significant between the two genotypes. Data is presented as mean ± SE. *P < 0.0001. For each genotype at each time point, *n* ≥ 3. **b** Sholl analysis of microglial morphology showing the numbers of processes crossing the 40-μm grid line (complete data shown in Additional file 1: Figure S1) for AIF1-positive cells in both crushed and contralateral control retinas of each genotype. Wild-type microglia exhibited an increase in processes at 7 days after crush (*P < 0.0001 relative to contralateral wild-type retinas), while microglia in Bax-deficient mice exhibited a significant decrease in processes by 14 days (**P < 0.0001, relative to control retinas). By 21 days, both control and crush retinas of each genotype exhibited similar levels of ramification, which were significantly reduced compared to earlier time points (*P* < 0.005). Data is presented as mean ± SE. A minimum of 50 cells from at least three mice, at each time point, were measured

The QPCR data was supported by immunolabeling in retinas for AIF1 protein after optic nerve crush (Fig. 2). In wild-type mice (Fig. 2a–d), AIF1 positive cells were sparsely detected in the uncrushed contralateral eye, and the microglia present were small with branched processes, characteristic of resting “ramified” microglia [21, 29]. At 7 and 14 days, a population of AIF1-positive cells was abundant in the ganglion cell layer (GCL) and the associated nerve fiber layer (NFL), inner nuclear layer (INL), and outer nuclear layer (ONL). By 21 days after injury, AIF1 labeling decreased throughout the retina in wild-type mice, but still exhibited more labeling than the contralateral eye. The *Bax*^−/−^ mice showed a similar baseline expression of AIF1 in the retina; however, after crush, the level of AIF1 labeling was markedly reduced at 7, 14, and 21 days relative to the wild-type mice (Fig. 2e–h). The morphology of AIF1-expressing cells in the NFL was also examined in whole-mounted retina preparations (Fig. 3). In wild-type mice at 7 days after injury, the numbers of cells appeared to increase and then decrease by 14 and 21 days (Fig. 3b–d). Crush injury did not appear to alter the density of the microglia in *Bax*^−/−^ mice (Fig. 3e–h). Quantification of cell density indicated that by 7 days post-crush, wild-type retinas showed a significant increase in the density of microglial cells in the NFL of crushed retinas (*P* < 0.0001 relative to contralateral eyes), which persisted through 14 days after injury (Fig. 4a). The density of AIF1-positive microglia peaked at 7–14 days post-crush, consistent with the peak in mRNA expression at 7 days (Fig. 1) and other studies [11]. By 21 days, the density of microglia was still significantly higher than the contralateral eye, but the numbers were declining. Unlike wild-type mice, *Bax*-deficient mice exhibited only a modest increase in microglial density, although the density of microglia gradually rose in both the injured and contralateral retinas of the knockout mice. Importantly, *Bax*^−/−^ mice exhibited a significantly lower density of microglia, relative to injured retinas of wild-type mice, at both 7 and 14 days post-crush (*P* < 0.0001). Interestingly, control retinas of both *Bax*^+/+^ and *Bax*^−/−^ mice showed a modest increase in microglial density over the time course examined, indicative of a contralateral eye effect from optic nerve injury, which has been described by others [82, 83].

A Sholl analysis was also conducted to quantify morphological changes in these cells after optic nerve damage. Quantification of retinas in each genotype, at different days, is shown in Additional file 1: Figure S1A–F. Figure 4b shows data for all genotypes at all days for processes crossing the 40-μm grid line. Cells in wild-type retinas show a significant increase in processes 7 days after optic nerve damage (compared to contralateral eyes, *P* < 0.001), while at 14 and 21 days, cells in both retinas had similar morphology, which was overall reduced in complexity relative to their shape at 7 days (*P* < 0.0005). Cells in *Bax*^−/−^ retinas showed no change between eyes at 7 days after crush, but had significantly fewer processes in the damaged eyes by 14 days (*P* < 0.001). As with the wild-type retinas, there was an overall reduction in the numbers of processes in both eyes at 14 and 21 days, compared to 7 days (*P* < 0.005).

Morphologically, the microglia in the uncrushed retinas appeared different between the two genotypes. Wild-type mice exhibited a range of morphologies for microglia varying from cells with thick cell bodies and large processes to cells with small somas and highly ramified fine processes. While a similar distribution was also evident in the *Bax*^−/−^ mice, a greater proportion of cells appeared to have thick cell bodies and large processes. The average numbers of processes as assessed by the Sholl analysis (see Fig. 4b), and the average area of cells between the two genotypes, were not significantly different (*P* = 0.71 and 0.32, respectively). However, scoring of images for amoeboid-like or ramified-like morphologies showed that wild-type mice presented with ~58 % ramified-like microglia, while *Bax*^−/−^ animals presented with ~60 % amoeboid-like cells, which was significantly different (*P* = 0.003).

**Fig. 5:**
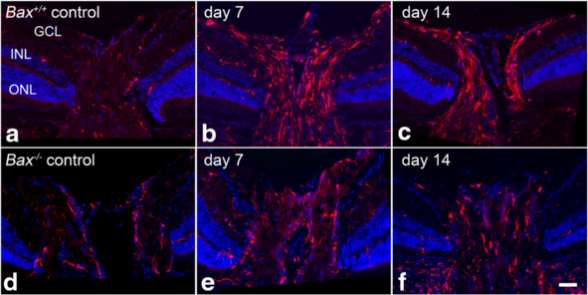
Microglial activation is present but reduced in the optic nerve head of *Bax* ^−/−^ mice after crush. The microglial populations in the optic nerve heads of *Bax* ^+/+^ and *Bax* ^−/−^ mice were also compared. **a**–**c** Wild-type mice exhibited a clear increase in the intensity and number of AIF1-expressing cells (*red label*) at both 7 and 14 days after crush. **d**–**f** Unlike in the retina, mice deficient for *Bax* also exhibited an increase in microglia at the optic nerve head 7 and 14 days post-crush, although the number of cells and intensity did not appear as high as the wild-type counterparts. DAPI nuclear counterstain (*blue label*). *Scale bar* 50 μm. For each genotype at each time point, *n* ≥ 3

The microglial response in the optic nerve head of the *Bax*^+/+^ and *Bax*^−/−^ mice was also noticeably different. In control eyes, similar levels of AIF1-positive cells populated the ONH of both genotypes (Fig. 5a, d). In the wild-type mice, crush injury caused the number of microglia and intensity of AIF1 staining to increase at 7 and 14 days (Fig. 5b, c). In contrast, in the *Bax*^−/−^ mice the amount of AIF1 labeling was quite attenuated despite an increase in the number of microglia in the injured relative to the control optic nerve head, compared to wild-type mice (Fig. 5e, f).

### Macroglial activation is attenuated in *Bax*^−/−^ mice after optic nerve crush

**Fig. 6:**
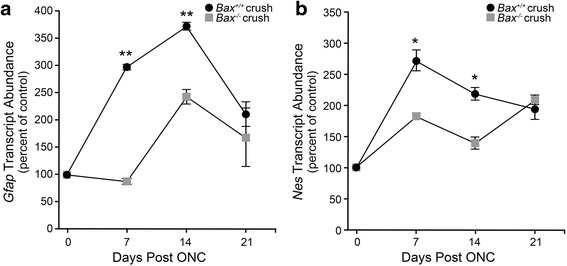
Expression of macroglial markers *Gfap* and *Nes* are significantly attenuated after crush in *Bax* ^−/−^ mice. Wild-type and *Bax* ^−/−^ mice were evaluated for macroglial activation markers following optic nerve crush. **a** In wild-type mice, *Gfap* mRNA expression rose sharply by 7 days, and peaked at 14 days before declining again at 21 days post-crush. The *Bax* ^−/−^ mice, however, showed no change in *Gfap* expression 7 days after crush, and although expression was elevated by 14 days, it still remained significantly lower than the wild-type mice. By 21 days, both genotypes were trending towards baseline and were no longer significantly different. **b** A second macroglial activation marker, *Nes*, was also elevated in wild-type mice by 7 days after crush, after which expression steadily declined at 14 and again 21 days. Although *Bax* ^−/−^ mice also exhibited a rise in *Nes* expression at 7 and 14 days post-crush, the values were significantly lower than those seen in wild-type mice. By 21 days *Nes* expression levels were no longer significant between the wild-type and knockout mice. Data is presented as mean ± SD. *P < 0.001, **P < 0.0001. For each genotype at each time point, *n* ≥ 3

Mice deficient for *Bax* also exhibited a reduction in the expression of macroglial activation markers after optic nerve crush. As shown in Fig. 6a, at 7 days after crush, *Gfap* expression in the wild types was significantly elevated above the contralateral eye (*P* < 0.0001), while the injured retinas of *Bax*^−/−^ mice showed only a modest increase that was greatly attenuated compared to wild-type mice (*P* < 0.0001). By 14 days, macroglial activation increased in both genotypes, however expression in the wild type mice still remained significantly elevated over *Bax*^−/−^ mice (*P* < 0.0001). By 21 days, *Gfap* expression began to trend towards baseline levels in both genotypes, and no longer retained statistical significance (*P* = 0.28). A second macroglial activation marker, Nestin (*Nes)* [24, 26, 27, 84], was also analyzed by QPCR. Similar to *Gfap*, the expression of *Nes* at 7 and 14 days post-crush was attenuated in the crushed retinas of *Bax*^−/−^ mice relative to the levels observed in the crushed wild-type retinas (Fig. 6b, *P* < 0.001). By 21 days, expression continued to decline in the wild-type mice, but rose slightly in the *Bax*^−/−^ mice, although the expression levels were no longer statistically different between the two genotypes (*P* = 0.37).

**Fig. 7:**
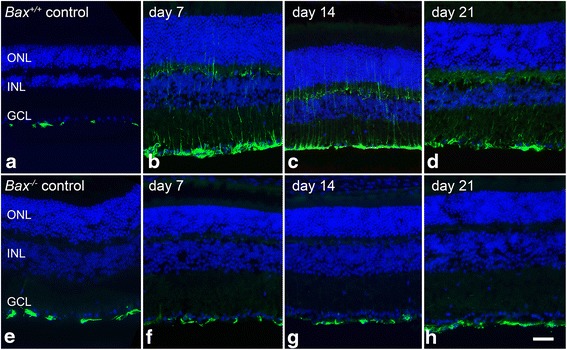
Müller cell upregulation of GFAP is absent after crush in *Bax* ^−/−^ mice. The expression of GFAP protein (*green label*) was also evaluated in retinal sections from wild-type and *Bax* ^−/−^ mice after crush. **a**–**d** Astrocytes expressed a baseline level of GFAP in the retinas of wild-type mice, and by 7 days after crush, there was a dramatic increase in GFAP labeling in the ganglion cell layer (GCL), as well as through the retinal layers, indicative of Müller cell activation. Müller cells continued to express GFAP at 14 and 21 days, although expression levels appeared to decline at the latter time point. **e**–**h** While retinas of *Bax* ^−/−^ mice also expressed GFAP in the astrocytes of control eyes, after crush, there was no upregulation of GFAP in the GCL, or through the retinal layers. Inner nuclear layer (INL), outer nuclear layer (ONL). DAPI nuclear counterstain (*blue label*). *Scale bar* 50 μm. For each genotype at each time point, *n* ≥ 3

Immunofluorescent labeling for GFAP performed on retinal sections showed that astrocytes expressed a baseline level of GFAP in the NFL of both *Bax*^+/+^ and *Bax*^−/−^ mice (Fig. 7a, e). At 7 days after crush, the GFAP labeling intensified in the wild-type mice and fibers appeared through the retinal layers indicative of Müller cell expression. This expression pattern of GFAP persisted in the wild-type mice at 14 and 21 days, although at the latter time point, GFAP expression was markedly reduced. The *Bax*^−/−^ retinas contrasted starkly with the wild-type mice at all time points analyzed (Fig. 7e–h). There was a complete absence of GFAP labeling in the Müller cells, resulting in a labeling pattern that did not appear to differ from the control eyes.

### Microglial and macroglial activation are not dependent on BAX

**Fig. 8:**
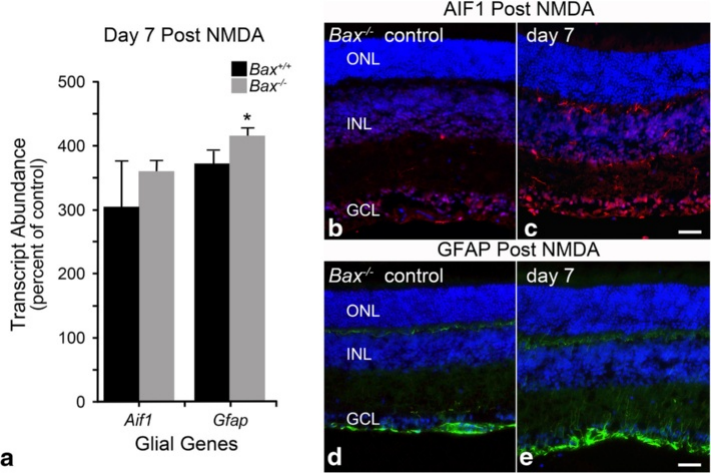
NMDA treatment triggers microglial and macroglial activation in *Bax* ^−/−^ mice. Wild-type and *Bax* ^−/−^ mice received an intraocular injection of NMDA to confirm that *Bax-*deficient glia were capable of mounting an activation response. Mice were evaluated for microglial and macroglial activation 7 days post-injection. **a** Both wild-type and *Bax* ^−/−^ mice exhibited a significant increase in the expression of *Aif1* and *Gfap* relative to control eyes. The values for *Aif1* expression were not statistically different between the two genotypes, although *Gfap* expression was significantly higher in *Bax* ^−/−^ mice relative to the wild-type mice (*P < 0.05). **b**, **c** Immunofluorescence also revealed a clear increase in AIF1 labeling (*red*) in the retinas of *Bax* ^−/−^ mice, with microglia appearing in the ganglion cell layer (GCL) and inner and outer plexiform layers. **d**, **e** The expression of GFAP (*green label*) was also confirmed in *Bax* ^−/−^ mice. A clear increase in labeling was seen in the GCL, as well as through the retinal layers, indicating that *Bax* ^−/−^ Müller glia are capable of upregulating GFAP. **a** Data is presented as mean ± SD. Inner nuclear layer (INL), outer nuclear layer (ONL). DAPI nuclear counterstain (*blue label*). *Scale bar* 50 μm. For each genotype, *n* ≥ 3

The attenuated glial response in *Bax*-deficient mice supports the hypothesis that RGC soma death is an important event in the signaling of retinal glial activation. To strengthen this interpretation, we next examined if retinal glia in *Bax*^−/−^ mice could become activated using an alternative damage paradigm. While BAX has not been linked to glial activation, we wanted to confirm that *Bax-*deficient glia were capable of upregulating *Aif1* and *Gfap*. In this experiment, we introduced the glutamate analog *N*-*methyl*-d-aspartate (NMDA) by an intravitreal injection, which has previously been shown to kill RGCs in both wild-type and *Bax*-deficient mice [50]. NMDA has also been reported to directly interact with NMDA receptors expressed by Müller cells [85]. Examination of both transcript levels and protein immunostaining showed a predictable microglial and macroglial response in both genotypes. By 7 days post-NMDA injection, *Aif1* and *Gfap* mRNA expression were significantly elevated in both genotypes of mice (Fig. 8a, *P* < 0.0001 relative to contralateral eyes). Immunofluorescent labeling also revealed a clear increase in AIF1-positive cells in the GCL and plexiform layers of *Bax*^−/−^ retinas (Fig. 8b, c), as well as an increase in GFAP labeling in the GCL and through the retinal layers, indicating Müller cell activation (Fig. 8d, e). Wild-type mice exhibited similar changes in protein expression following NMDA treatment (data not shown).

### ATP analogs trigger an increase in macroglial activation markers

**Fig. 9:**
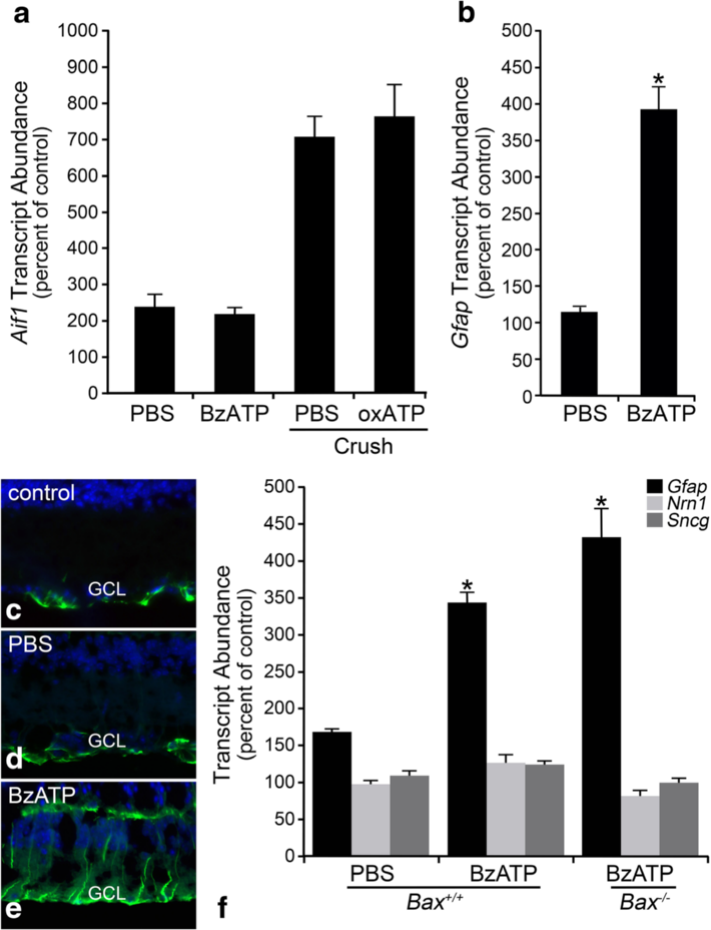
A P2X receptor agonist triggers macroglial activation without inducing RGC injury. **a** Microglial activation was evaluated by monitoring *Aif1* expression 48 h after an intraocular injection of BzATP. This treatment was not found to affect *Aif1* mRNA levels relative to PBS-treated retinas (*P* = 0.21). Additionally, when mice were pre-treated with an intraocular injection of oxATP, a P2X receptor antagonist, prior to optic nerve crush, by 7 days post-crush *Aif1* expression by the microglial population not statistically different from PBS-injected retinas (*P* = 0.20). Data is presented as mean ± SD. For each treatment group, *n* ≥ 3. **b** Wild-type mice were treated with an intraocular injection of the P2X receptor agonist, BzATP, which induced a clear increase in *Gfap* mRNA expression by 48 h. **c**–**e** Immunofluorescence of retinal sections confirmed that BzATP triggered an increase in GFAP expression (*green label*), most obviously in the Müller cell population. The PBS-treated eyes did not exhibit an increase in GFAP. DAPI nuclear counterstain (*blue label*). *Scale bar* 50 μm. **f** The change in *Gfap* expression after BzATP treatment was also evaluated in *Bax* ^−/−^ mice. By 48 h, *Gfap* expression was elevated in both wild-type and knockout mice, indicative of functional purinergic receptors on *Bax*-deficient glia. Importantly, markers of RGC injury (*Sncg* and *Nrn1*) did not decline, indicating glial activation occurred in the absence of RGC injury. Data is presented as mean ± SD. Ganglion cell layer (GCL). *P < 0.005. For each treatment group and genotype, *n* ≥ 3

There is growing evidence that injured neurons release ATP as a distress signal into the extracellular space, so we next wanted to determine if ATP analogs triggered glial activation in the retina. An intraocular injection of BzATP, an agonist selective for P2X receptors was delivered to wild-type mice, and microglial and macroglial activation markers were analyzed 48 h later. RGC gene expression was also monitored as a sign of injury, as previous studies have shown that this agonist can trigger RGC degeneration [75, 76]. While BzATP had no effect on inducing *Aif1* expression in microglia (Fig. 9a), this agonist triggered macroglial activation as indicated by a clear rise in *Gfap* mRNA expression (Fig. 9b), and an increase in GFAP protein labeling, particularly in the Müller glia (Fig. 9c–e). BzATP was also delivered to *Bax*^−/−^ mice to confirm the functionality of purinergic receptors in the absence of BAX. By 48 h, *Gfap* expression was significantly elevated in the injected eye of both wild-type and *Bax*^−/−^ mice, relative to the contralateral eye (Fig. 9f). To determine if P2X receptor activation affected the RGCs, and cause indirect macroglial activation, we evaluated if it resulted in a decrease in RGC-specific gene expression, which is a sensitive indicator of damage to these cells [54, 86–88]. BzATP had no effect on mRNA levels of *Nrn1* or *Sncg* (Fig. 9e), suggesting that RGCs were not adversely affected by this agonist, within the 48-h time frame evaluated in this experiment. This observation, added to the lack of microglial activation, indicated that BzATP was acting principally on the macroglia.

### A P2X receptor antagonist limits, but does not prevent, macroglial cell activation after optic nerve crush

**Fig. 10:**
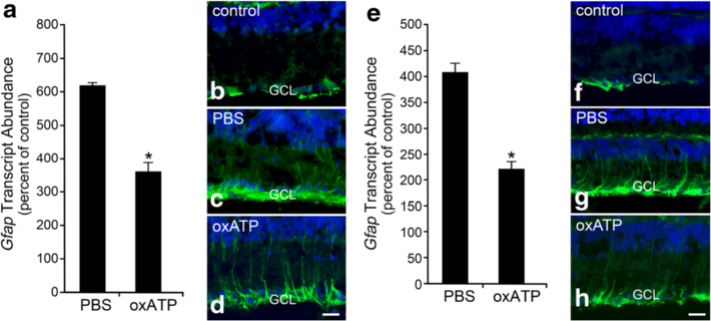
Treatment with oxATP reduces *Gfap* mRNA expression after crush injury. To evaluate purinergic signaling in the crush paradigm, wild-type mice were treated with oxATP either prior to or after optic nerve crush, and evaluated for macroglial expression of *Gfap* 7 days after injury. **a** Treatment with oxATP 1 day prior to optic nerve crush reduced *Gfap* transcript levels by about 50 %, relative to the PBS-injected eyes. **b**–**d** Despite the decline in *Gfap* mRNA levels, the upregulation of GFAP protein by Müller cells was not inhibited with oxATP pre-treatment. **e** A post-treatment of oxATP delivered 3 days after crush also reduced *Gfap* expression by about 50 %. **f**–**h** The post-treatment was also ineffective at reducing Müller cell activation, as fibers of GFAP were still clearly present through the retinal layers, and did not appear dramatically different than the PBS-treated retinas. Interestingly, oxATP in both treatment groups appeared to reduce the intensity of GFAP expression in the nerve fiber layer, suggesting oxATP may selectively affect astrocyte expression of the filament protein in the crush paradigm. **a**, **e** Data is presented as mean ± SD. *P < 0.001. *Scale bar* 50 μm. For each treatment group, *n* ≥ 3 for quantitative analysis by QPCR, *n* = 3 for immunofluorescent labeling

While the previous results show that purinergic signaling can mediate activation of retinal macroglia, they do not verify if this mechanism of signaling is active in the optic nerve crush paradigm of damage. To test this, we examined if a P2X receptor antagonist (oxATP) was able to block macroglial activation after crush. We delivered an intraocular injection of the drug 24 h prior to, or 3 days post-crush, and analyzed for glial activation 7 days after surgery when peak activation is known to occur. The expression of *Gfap* was significantly elevated in the crushed eye of all treatment groups (Fig. 10, *P* < 0.0005, relative to the contralateral eye); however, both pre-treating and post-treating, mice with an intraocular injection of oxATP attenuated *Gfap* expression by about half (Fig. 10a, e). Immunostaining in retinal sections showed that neither pre- nor post-crush treatment with oxATP was able to completely block GFAP expression in Müller cells, while there was a marked decrease in apparent staining in the NFL (Fig. 10b–d and f–h), suggesting that the 50 % decrease in *Gfap* expression may be attributable to astrocytes. Consistent with no effect of BzATP on activating *Aif1* expression in microglia, oxATP had no effect on suppressing Aif1 expression after optic nerve crush (Fig. 9a).

### *Panx1* deficiency in RGCs does not prevent macroglial activation after crush

It appears that purinergic signaling may play a role in triggering *Gfap* upregulation after crush, although the source of extracellular ATP in response to optic nerve damage is not known. We hypothesized that ATP was released from RGCs undergoing apoptosis, and wanted next to explore the mechanism by which ATP release may occur. One possible mechanism of ATP release involves the PANX1 hemichannel, which is expressed across the retinal cell types and is highly enriched in RGCs [89]. This mechanism is consistent with the attenuated glial response observed in *Bax*^−/−^ mice, since dying cells are known to release ATP through caspase 3/7-mediated activation of the PANX1 protein [60, 90]. Since BAX activation is required for activation of caspases, it is reasonable to predict that *Bax*-deficient RGCs would not be able to activate PANX1 and release ATP. To test for whether glial activation is dependent on *Panx1* after injury, the *Panx1* gene was selectively disabled in either RGCs or all cell types in *Panx1-LoxP* mice using cell type-specific expression of CRE recombinase (see the “Methods” section).

**Fig. 11:**
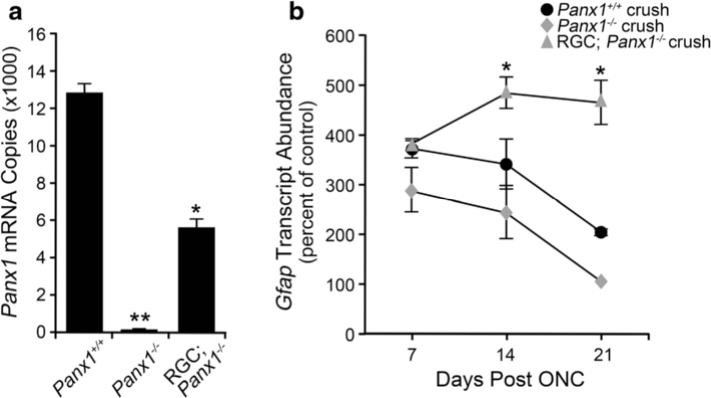
Genetic ablation of *Panx1* from RGCs does not reduce macroglial activation after optic nerve crush. We hypothesized that PANX1 hemichannels mediated ATP release from dying RGCs, and we examined the role of this protein in the crush paradigm by using mice genetically deficient for *Panx1* in either RGCs or all cell types. **a** QPCR analysis showed that, relative to wild-type mice, total knockout mice expressed undetectable levels of *Panx1*, while RGC conditional knockout mice exhibited a 50 % reduction in mRNA expression. **b** Wild-type and *Panx1*-deficient mice were then subjected to crush and analyzed for macroglial activation. Wild-type mice exhibited characteristic *Gfap* expression, with levels peaking at 7 days before declining at 14 and 21 days. Similarly, at 7 days in the *Panx1* ^−/−^ mice, *Gfap* mRNA abundance was highest, and gradually declined at 14 and 21 days. Mice deficient for *Panx1* in RGCs exhibited sustained levels of macroglial activation as *Gfap* expression rose between 7 and 14 days, and remained significantly elevated above wild-type mice at 21 days post-crush. Data is presented as mean ± SD. *P < 0.0005, **P < 0.0001. For each genotype at each time point, *n* ≥ 3

**Fig. 12:**
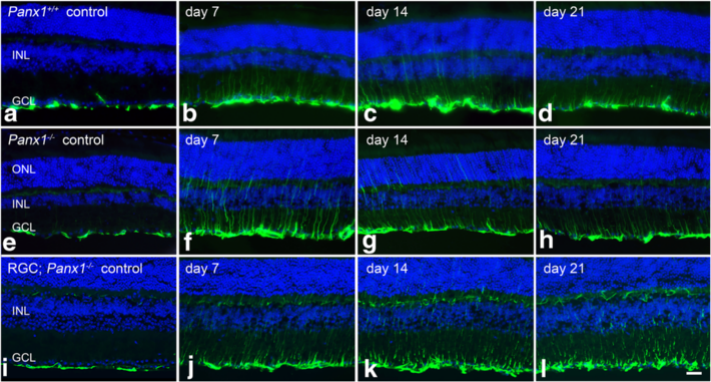
Müller cell upregulation of GFAP after optic nerve crush is not affected by *Panx1* deficiency. Wild-type and *Panx1* conditional knockout mice were evaluated for GFAP expression after optic nerve crush. Genetic ablation of *Panx1* did not appear to alter astrocyte expression of GFAP in control eyes (**a**, **e**, **i**). At 7, 14, and 21 days post-crush, all genotypes exhibited a clear upregulation of GFAP, indicated by an increase in intensity in the nerve fiber layer, and through the retinal layers, consistent with Müller cell activation. GFAP expression appeared strongest between 7 and 14 days, although Müller cell expression of GFAP was still present 21 days after crush injury. There did not appear to be an obvious difference in the pattern of macroglial activation between the three genotypes. *Scale bar* 50 μm. For wild-type and *Panx1* ^*−*/*−*^ mice, at each time point *n* ≥ 3. For *Panx1* ^*−*/*−*^ RGC mice, 7 days *n* = 3, and at 14 and 21 days *n* = 2

The retinas were first evaluated for *Panx1* mRNA by QPCR, which showed a complete abrogation of *Panx1* expression in *Panx1*^−/−^ mice, while RGC *Panx*^−/−^ retinas showed about a 50 % reduction in mRNA expression (Fig. 11a). Conditional knockout and wild-type mice were then subjected to optic nerve crush and evaluated for changes in macroglial *Gfap* expression. Wild-type mice and *Panx1*^−*/*−^ mice followed similar trends in *Gfap* expression, with levels peaking at 7 days and then declining at 14 days and again at 21 days (Fig. 11b). The RGC *Panx1*^−*/*−^ mice, however, consistently exhibited higher and sustained *Gfap* expression after crush relative to wild-type and *Panx1*^−/−^ mice. While *Gfap e*xpression was not statistically different from wild-type mice at 7 days, the mRNA levels peaked at 14 days and remained elevated at 21 days relative to wild type and RGC *Panx1*^−*/*−^ mice (*P* < 0.0005). Retinal sections were further evaluated by immunolabeling, which showed an increase in GFAP expression in the nerve fiber layer and through the retinal layers, consistent with astrocyte and Müller cell activation (Fig. 12). The increase in GFAP was present in all genotypes, and at all post-crush time points examined, while the control eyes only exhibited baseline levels of GFAP by astrocytes. GFAP labeling appeared to be most prominent at 7 and 14 days, although Müller cell activation was still prevalent by 21 days.

## Discussion

### The timing of RGC pathology and glial activation after optic nerve damage

An understanding of the sequence of events being executed in damaged RGCs is relevant to understanding how retinal glia respond. We have monitored the glial response using the markers *Aif1* and *Gfap*. We previously determined that mRNA levels for these markers become elevated between 3 and 5 days after crush [55]. Importantly, both glial populations appeared to be synchronized with RGC pathology that followed the interval of BAX protein activation, which is estimated to occur by 3 days after acute optic nerve damage [91, 92] (M. Maes and R. Nickells, unpublished data), suggesting that apoptotic events downstream of the BAX-dependent step were linked to the signaling mechanism leading to the glial response. This, in fact, appears to be the case, since both microglial and macroglial responses are significantly suppressed in the absence of RGC death in *Bax*-deficient mice. A caveat in our study is that microglial and macroglial activation rose modestly in the injured and contralateral retinas of both genotypes. Additional activation signals, while minimally influential, may be released prior to the *Bax*-dependent step of the apoptotic program, or originate from a source other than dying RGCs.

### Potential role of purinergic signaling between injured RGCs and retinal glia

Given the prerequisite for RGC death and the timing of the glial response, we reasoned that RGCs could be communicating with the glia via purinergic signaling pathways. We show that an injection of BzATP into the mouse eye stimulates *Gfap* expression in retinal macroglia, including Müller cells. This is particularly intriguing, since BzATP is historically considered a specific agonist for the P2X7 receptor [93], and there is conflicting evidence about the expression of this receptor by Müller cells [94–96]. How then, do the Müller glia respond to BzATP? It is possible that different purinergic receptors on Müller cells are responding to the agonist, especially at the relatively high concentrations that were used for intravitreal injection. BzATP is a similarly potent agonist for the P2X1 receptor, and has partial activity for multiple P2X and P2Y1 receptors [97–99]. While there is evidence that P2X7R is not expressed by mammalian Müller cells, there are reports that Müller cells in the lower vertebrate species do express this receptor [96]. Additionally, we cannot rule out that this receptor is not upregulated in Müller cells under stress conditions [100]. Lastly, it may be possible that the Müller cell response was secondary to the action of BzATP on either RGCs or astrocytes, which do contain P2X7 receptors [94, 101]. Activation of P2X7R on other cell types may lead to ATP release that then affect the Müller cells via a different purinergic receptor [102]. Experiments using P2X antagonists shed little light on this mechanism. While we show that oxATP, also considered a relatively selective antagonist for the P2X receptors [103], can partially suppress the macroglial response, this does not rule out a secondary activating mechanism from the astrocytes or RGCs to the Müller cells. Additionally, suppression of glial activation using oxATP appears to selectively affect the response in astrocytes when assessed by immunostaining. Further studies are necessary to help elucidate the purinergic signaling network in the retina after optic nerve damage.

While purinergic signaling appears to play a role in macroglial activation, our data do not show an effect on microglial activation, at least in the context of upregulating *Aif1* expression. Microglia reportedly express the P2X7 receptor [104, 105], and activation of this receptor has been directly linked to microglial inflammatory responses in rat primary hippocampal cultures [71]. Nevertheless, experiments using both BzATP and oxATP appear to exclude purinergic signaling to activate these cells after optic nerve damage, and it is not possible to rule out an effect on the microglia based on other metrics typical of their activation response. It is clear, however, that *Bax*-deficient mice exhibit a suppression of microglial activation in this damage paradigm, which is consistent with a recent study using an acute ethanol toxicity model, in which microglial activation was also suppressed in *Bax*^−/−^ mice [106]. The signaling pathway linking RGCs and microglia was not a focus of this study, but we hypothesize that it likely occurs via Toll-like receptors (TLRs). This is supported by three lines of experimental evidence. Retinal microglia upregulate TLR expression as an early response to glaucomatous damage [107], polymorphisms in both *TLR2* and *TLR4* are risk alleles for glaucoma [108, 109], and deletion of *Tlr4* in mice reduces RGC death after optic nerve damage [110] and ischemia-reperfusion [111]. TLRs respond to endogenously derived “damage-associated molecular pattern” (DAMP) molecules released from damaged or dying cells [3]. For example, TLR9 additionally recognizes mitochondrial DNA [112], which would only be released from cells undergoing mitochondrial breakdown, a feature expected only in cells with a functional BAX protein. Perhaps, not coincidentally, this receptor has also been linked to glial activation in the retina [113].

One noteworthy observation in our study involving the microglia response in wild-type mice after optic nerve damage is the increase in ramifications of these cells at 7 days after injury. The classic activation response of microglia is to convert to a more amoeboid state by the retraction of processes, after which they enter a transitional/motile phenotype by extending new processes [21]. It is possible that our observations at 7 days correspond to cells already in this transitional phase.

### PANX1 function in RGCs after optic nerve damage

Based on the suppressed glial response in Bax-deficient mice, and the pharmacologic evidence suggesting a role for purinergic signaling in macroglial activation, we hypothesized that ATP was released from dying RGCs via activated PANX1 channels. This hypothesis was particularly attractive given that mechanical stress applied to RGCs elicits ATP release via PANX1 hemichannels [65]. Additionally, activated caspases proteolytically activate PANX1 [60]; therefore, PANX1 activation would not occur in *Bax*-deficient cells, which fail to stimulate caspases. However, *Panx1* ablation in RGCs failed to reduce the macroglial response invalidating our hypothesis. Several lines of evidence implicate PANX1 as playing a role in RGC pathology, although none of these studies evaluated the effects after optic nerve damage. In ischemia damage models, deletion of *Panx1* in RGCs provides a protective response [78], including attenuation of neurotoxic Ca^2+^ uptake. *Panx1*^−/−^ RGCs also exhibited impaired activation of the inflammasome. Tied with this is evidence that prolonged exposure to ATP analogs, including BzATP, stimulates RGC loss [75, 76], presumably through the interaction of P2X7 receptors and PANX1 [114] on RGCs. In preliminary experiments, however, we did not detect a similar protective effect for RGCs after optic nerve damage when *Panx1* was selectively deleted in these neurons (data not shown). Conversely, optic nerve crush in the eyes with *Panx1*^−/−^ RGCs resulted in what appeared to be an elevated and sustained macroglial response, measured as a function of *Gfap* expression. This argues for a role of PANX1 channels in paracrine signaling between RGCs and activated macroglia to modulate or shutdown the activation response, although the nature of this paracrine response is yet to be determined.

A major caveat to our observations is that the sustained activation of macroglia after crush was not observed in the complete *Panx1*^−/−^ mouse as well. Currently, we have not been able to reconcile these conflicting results. One possible explanation, however, is the developmental timing of the *Panx1* deletion. In complete (zygotic) knockout mice generated by crossing animals with a floxed *Panx1* allele to the CMV-*Cre* transgenic line, the disabling of *Panx1* alleles occurs as early as the 1-2 cell stage of development [115]. Under these conditions, it is conceivable that *Panx1*^−/−^ mice develop compensatory mechanisms to overcome the loss of PANX1 hemichannels. Consistent with this, multiple functions attributed to *Panx1* have only been observed in mice with both *Panx1* and *Panx2* deleted [116]. In contrast, RGC-specific deletion of *Panx1* by AAV2-Cre injection is achieved well into adulthood, thereby excluding the development of a compensatory mechanism in these cells. We posit that these experiments more accurately reflect a role for *Panx1* in these cells.

### Glial cell representation and morphology in *Bax*-deficient mice

We used *Bax*^−/−^ mice to assess the relationship between RGC death and retinal glial activation, principally because RGCs lacking this proapoptotic member of the *Bcl2* gene family exhibit complete and sustained prevention of RGC loss in optic nerve damage paradigms [50–54]. It is relevant, however, to consider how the loss of *Bax* affects the glial population in our interpretation of the results of this study. This is especially important, given the well-documented phenomenon that *Bax*-deficiency leads to supernumerary numbers of some classes of neurons in the mouse [117–119]. Surprisingly, there is very little information regarding glial populations in the CNS of *Bax*-deficient mice. White and colleagues [118] found no evidence of reduced cell death of glial populations in these mice, presumably because they also express a functional proapoptotic BAK protein, unlike neurons, which alternatively splice the *Bak* transcript [120]. Conversely, oligodendrocyte cell density in the optic nerves of *Bax*-deficient mice is greater, retaining a normal ratio with the increased axon number [121]. Our comparison of microglial density in the peripheral retinal nerve fiber layers of wild-type and *Bax*-deficient mice suggests that there is no increase in microglial cells commensurate with the reported increase in RGCs [50, 122, 123]. The microglial population in the retinas of *Bax*-deficient mice is different from wild-type littermates, however, and appears to be enriched in cells with more amoeboid-like characteristics. The reason for this enrichment is not known, but perhaps *Bax* deficiency provides a bias towards a subclass of microglia [124] by preventing programmed cell death during development, similar to the phenomenon exhibited by neuronal populations. Importantly, however, our experiments using the excitotoxic agent NMDA to induce RGC death indicate that both microglia and macroglia in *Bax*^−/−^ mice were able to upregulate markers consistent with a normal activation response.

## Conclusions

Glial activation is associated with the neuroinflammatory response of many neurodegenerative conditions, but the signaling pathways eliciting this process are not fully defined. Here, we show that neuronal death appears to be a prerequisite for the full activation response observed in the retina after optic nerve damage, and that this response is modulated by purinergic signaling. This helps explain why *Bax*-deficient RGCs are completely resistant to optic nerve damage, even though the extrinsic apoptotic pathways, which would be activated during neuroinflammation, remain intact in these cells.

## Additional file

Additional file 1: Figure S1. Summary of Sholl analysis of AIF1-expressing microglia after optic nerve crush. (A–C) Wild-type retinas at 7, 14, and 21 days after optic nerve crush, respectively. (D–F) Bax^−/−^ retinas at 7, 14, and 21 days, respectively. Data is presented as mean ± SE. **P* < 0.05. Each graph represents a minimum of 50 cells measured from three retinas of each genotype. (JPG 2.19 mb)

## Abbreviations

AIF1: allograft inflammatory factor 1
ATP: adenosine triphosphate
BzATP: 2′(3′)-O-(4-benzoylbenzoyl)adenosine 5′-triphosphate triethylammonium salt
CD68: cluster of differentiation 68/macrosialin
GFAP: glial fibrillary acid protein
NES: nestin
NMDA: *N*-*methyl*-d-aspartate
oxATP: oxidized ATP
PANX1: pannexin 1
RGC: retinal ganglion cell

## References

[1] Nickells RW, Howell GR, Soto I, John SWM (2012). Under pressure: cellular and molecular responses during glaucoma, a common neurodegeneration with axonopathy. Ann Rev Neurosci.

[2] Quigley HA (2011). Glaucoma. Lancet.

[3] Soto I, Howell GR (2014). The complex role of neuroinflammation in glaucoma. Cold Spring Harb Perspect Med.

[4] Tezel G, Li LY, Patil RV, Wax MB (2001). TNF-alpha and TNF-alpha receptor-1 in the retina of normal and glaucomatous eyes. Invest Ophthalmol Vis Sci.

[5] Tezel G, Wax MB (2003). Glial modulation of retinal ganglion cell death in glaucoma. J Glaucoma.

[6] Yuan L, Neufeld AH (2001). Activated microglia in the human glaucomatous optic nerve head. J Neurosci Res.

[7] Zhang S, Wang H, Lu Q, Wang N, Wang Y, Yang D, Yan F (2009). Detection of early neuron degeneration and accompanying glial responses in the visual pathway in a rat model of acute intraocular hypertension. Brain Res.

[8] Bosco A, Steele MR, Vetter ML (2011). Early microglia activation in a mouse model of chronic glaucoma. J Comp Neurol.

[9] Fitzgerald M, Bartlett CA, Harvey AR, Dunlop SA (2010). Early events of secondary degeneration after partial optic nerve transection: an immunohistochemical study. J Neurotrama.

[10] Wang X, Tay SS-W, Ng Y-K (2000). An immunohistochemical study of neuronal and glial cell reactions in retinae of rats with experimental glaucoma. Exp Brain Res.

[11] Liu S, Li ZW, Weinreb RN, Xu G, Lindsey JD, Ye C, Yung WH, Pang CP, Shun D, Lam C (2012). Tracking retinal microgliosis in models of retinal ganglion cell damage. Invest Ophthalmol Vis Sci.

[12] Perez VL, Saeed AM, Tan Y, Urbieta M, Cruz-Guilloty F (2013). The eye: a window to the soul of the immune system. J Autoimmun.

[13] Inman DM, Horner PJ (2007). Reactive nonproliferative gliosis predominates in a chronic model of glaucoma. Glia.

[14] Garcia M, Vecino E (2003). Role of Muller glia in neuroprotection and regeneration in the retina. Histol Histopathol.

[15] Goldman D (2014). Muller glial cell reprogramming and retina regeneration. Nat Rev Neurosci.

[16] Iandiev I, Biedermann B, Bringmann A, Reichel MB, Reichenbach A, Pannicke T (2006). Atypical gliosis in Muller cells of the slowly degenerating rds mutant mouse retina. Exp Eye Res.

[17] Pekny M, Pekna M (2014). Astrocyte reactivity and reactive astrogliosis: costs and benefits. Physiol Rev.

[18] Chen H, Weber AJ (2002). Expression of glial fibrillary acidic protein and glutamine synthetase by Muller cells after optic nerve damage and intravitreal application of brain-derived neurotrophic factor. Glia.

[19] Wohl SG, Schmeer CW, Witte OW, Isenmann S (2010). Proliferative response of microglia and macrophages in the adult mouse eye after optic nerve lesion. Invest Ophthalmol Vis Sci.

[20] Bosco A, Inman DM, Steele MR, Wu G, Soto I, Marsh-Armstrong N, Hubbard WC, Calkins DJ, Horner PJ, Vetter ML (2008). Reduced retinal microglial activation and improved optic nerve integrity with minocycline treatment in the DBA/2J mouse model of glaucoma. Invest Ophthalmol Vis Sci.

[21] Stence N, Waite M, Dailey ME (2001). Dynamics of microglial activation: a confocal time-lapse analysis in hippocampal slices. Glia.

[22] Qu J, Jakobs TC (2013). The time course of gene expression during reactive gliosis in the optic nerve. PLoS One.

[23] Tonari M, Kurimoto T, Horie T, Sugiyama T, Ikeda T, Oku H (2012). Blocking endothelin-B receptors rescues retinal ganglion cells from optic nerve injury through suppression of neuroinflammation. Invest Ophthalmol Vis Sci.

[24] Hol EM, Pekny M (2015). Glial fibrillary acidic protein (GFAP) and the astrocyte intermediate filament system in diseases of the central nervous system. Curr Opin Cell Biol.

[25] Pekny M, Pekna M (2004). Astrocyte intermediate filaments in the CNS pathologies and regeneration. J Pathol.

[26] Xue L, Ding P, Xiao L, Hu M, Hu Z (2010). Nestin, a new marker, expressed in Müller cells following retinal injury. Can J Neurol Sci.

[27] Xue LP, Lu J, Cao Q, Kaur C, Ling EA (2006). Nestin expression in Muller glial cells in postnatal rat retina and its upregulation following optic nerve transection. Neurosci.

[28] Tezel G, Hernandez MR, Wax MB (2001). In vitro evaluation of reactive astrocyte migration, a component of tissue remodeling in glaucomatous optic nerve head. Glia.

[29] Karperian A, Ahammer H, Jelinek HF (2013). Quantitating the subtleties of microglial morphology with fractal analysis. Front Cell Neurosci.

[30] Kim SU, De Vellis J (2005). Microglia in health and disease. J Neurosci Res.

[31] Kumar A, Pandey RK, Miller LJ, Singh PK, Kanwar M (2013). Muller glia in retinal innate immunity: a perspective on their roles in endophthalmitis. Crit Rev Immunol.

[32] Olson JK, Miller SD (2004). Microglia initiate central nervous system innate and adaptive immune responses through mulitple TLRs. J Immunol.

[33] Krizaj D, Ryskamp DA, Tian N, Tezel G, Mitchell CH, Slepak VZ, Shestopalov VI (2014). From mechanosensitivity to inflammatory responses: new players in the pathology of glaucoma. Curr Eye Res.

[34] Constantinescu CS, Tani M, Ransohoff RM, Wysocka M, Hilliard B, Fujioka T, Murphy S, Tighe PJ, Das Sarma J, Trinchieri G (2005). Astrocytes as antigen-presenting cells: expression of IL-12/IL-23. J Neurochem.

[35] Fitzgerald M, Bartlett CA, Evill L, Rodger J, Harvey AR, Dunlop SA (2009). Secondary degeneration of the optic nerve following parital transection: the benefits of lomerizine. Exp Neurol.

[36] Payne SC, Bartlett CA, Harvey AR, Dunlop SA, Fitzgerald M (2011). Chronic swelling and abnormal myelination during secondary degeneration after partial injury to a central nervous system tract. J Neurotrama.

[37] Payne SC, Bartlett CA, Harvey AR, Dunlop SA, Fitzgerald M (2012). Myelin sheath decompaction, axon swelling, and functional loss during chronic secondary degeneration in rat optic nerve. Invest Ophthalmol Vis Sci.

[38] Yoles E, Muller S, Schwartz M (1997). NMDA-receptor antagonist protects neurons from secondary degeneration after partial optic nerve crush. J Neurotrauma.

[39] Levkovitch-Verbin H, Quigley HA, Kerrigan-Baumrind LA, D’Anna SA, Kerrigan D, Pease ME (2001). Optic nerve transection in monkeys may result in secondary degeneration of retinal ganglion cells. Invest Ophthalmol Vis Sci.

[40] Levkovitch-Verbin H, Quigley HA, Martin KR, Zack DJ, Pease ME, Valenta D (2003). A model to study differences between primary and secondary degeneration of retinal ganglion cells in rats by parital optic nerve transection. Invest Ophthalmol Vis Sci.

[41] Levkovitch-Verbin H, Dardik R, Vander S, Melamed S (2010). Mechanism of retinal ganglion cell death in secondary degeneration of the optic nerve. Exp Eye Res.

[42] Baptiste DC, Powell KJ, Jollimore CAB, Hamilton C, LeVatte TL, Archibald ML, Chauhan BC, Robertson GS, Kelly MEM (2005). Effects of minocycline and tetracycline on retinal ganglion cell survival after axotomy. Neurosci.

[43] Levkovitch-Verbin H, Kalev-Landoy M, Habot-Wilner Z, Melamed S (2006). Minocycline delays death of retinal ganglion cells in experimental glaucoma and after optic nerve transection. Arch Ophthalmol.

[44] Tezel G, Yang X, Yang J, Wax MB (2004). Role of tumor necrosis factor receptor-1 in the death of retinal ganglion cells following optic nerve crush injury in mice. Brain Res.

[45] Bosco A, Romero CO, Breen KT, Chagovetz AA, Steele MR, Ambati BK, Vetter ML (2015). Neurodegeneration severity can be predicted from early microglia alterations monitored in vivo in a mouse model of chronic glaucoma. Dis Model Mech.

[46] Roh M, Zhang Y, Murakami Y, Thanos A, Lee SC, Vavvas DG, Benowitz LI, Miller JW (2012). Etanercept, a widely used inhibitor of tumor necrosis factor a (TNF-a), prevents retinal ganglion cell loss in a rat model of glaucoma. PLoS One.

[47] Nakazawa T, Nakazawa C, Matsubara A, Noda K, Hisatomi T, She H, Michaud N, Hafezi-Moghadam A, Miller JW, Benowitz LI (2006). Tumor necrosis factor-a mediates oligodendrocyte death and delayed retinal ganglion cell loss in a mouse model of glaucoma. J Neurosci.

[48] Yang Z, Zack DJ (2011). What has gene expression profiling taught us about glaucoma?. Exp Eye Res.

[49] Nickells RW, Pelzel HR (2015). Tools and resources for analyzing gene expression changes in glaucomatous neurodegeneration. Exp Eye Res.

[50] Li Y, Schlamp CL, Poulsen KP, Nickells RW (2000). Bax-dependent and independent pathways of retinal ganglion cell death induced by different damaging stimuli. Exp Eye Res.

[51] Libby RT, Li Y, Savinova OV, Barter J, Smith RS, Nickells RW, John SWM (2005). Susceptibility to neurodegeneration in glaucoma is modified by Bax gene dosage. PLoS Genet.

[52] Howell GR, Libby RT, Jakobs TC, Smith RS, Phalan FC, Barter JW, Barbay JM, Marchant JK, Mahesh N, Porciatti V (2007). Axons of retinal ganglion cells are insulted in the optic nerve early in DBA/2 J glaucoma. J Cell Biol.

[53] Semaan SJ, Li Y, Nickells RW (2010). A single nucleotide polymorphism in the Bax gene promoter affects transcription and influences retinal ganglion cell death. ASN Neuro.

[54] Janssen KT, Mac Nair CE, Dietz JA, Schlamp CL, Nickells RW (2013). Nuclear atrophy of retinal ganglion cells precedes the Bax-dependent stage of apoptosis. Invest Ophthalmol Vis Sci.

[55] Mac Nair CE, Fernandes KA, Schlamp CL, Libby RT, Nickells RW (2014). Tumor necrosis factor alpha has an early protective effect on retinal ganglion cells after optic nerve crush. J Neuroinflamm.

[56] Kielian T (2006). Toll-like receptors in central nervous system glial inflammation and homeostasis. J Neurosci Res.

[57] Rogove AD, Lu W, Tsirka SE (2002). Microglial activation and recruitment, but not proliferation, suffice to mediate neurodegeneration. Cell Death Differ.

[58] Rubartelli A (2014). DAMP-mediated ativation of NLRP3-inflammasome in brain sterile inflammation: the fine line betwen healing and neurodegeneration. Front Immunol.

[59] Sofroniew MV, Vinters HV (2010). Astrocytes: biology and pathology. Acta Neuropathol.

[60] Chekeni FB, Elliott MR, Sandilos JK, Walk SF, Kinchen JM, Lazarowski ER, Armstrong AJ, Penuela S, Laird DW, Salvesen GS (2010). Pannexin1 channels mediate ‘find-me’ signal release and membrane permeability during apoptosis. Nature.

[61] Chekeni FB, Ravichandran KS (2011). The role of nucleotides in apoptotic cell clearance: implications for disease pathogenesis. J Mol Med.

[62] Elliot MR, Chekeni FB, Trampont PC, Lazarowski ER, Kadl A, Walk SF, Park D, Woodson RI, Ostankovich M, Sharma P (2009). Nucleotides released by apoptotic cells act as a find-me signal to promote phagocytic clearance. Nature.

[63] Li A, Zhang X, Ge J, Laties AM, Mitchell CH (2011). Sustained elevation of extracellular ATP in aqueous humor from humans with chronic angle-closure glaucoma. Exp Eye Res.

[64] Reigada D, Lu W, Zhang M, Mitchell CH (2008). Elelvated pressure triggers a physiological release of ATP from the retina: possible role for pannexin hemichannels. Neurosci.

[65] Xia J, Lim JC, Lu W, Beckel JM, Macarak EJ, Laties AM, Mitchell CH (2012). Neurons respond directly to mechanical deformation with pannexin-mediated ATP release and autostimulation of P2X7 receptors. J Physiol.

[66] Beckel JM, Argall AJ, Lim JC, Xia J, Lu W, Coffey EE, Macarak EJ, Shahidullah M, Delamere NA, Zode GS (2014). Mechanosensitive release of adenosine 5′-triphosphate through pannexin channels and mechanosensitive upregulation of pannexin channels in optic nerve head astrocytes: a mechanism for purinergic involvement in chronic strain. Glia.

[67] Lu W, Hu H, Sévigny J, Gabelt BT, Kaufman PL, Johnson EC, Morrison JC, Zode G, Sheffield VC, Zhang X (2015). Rat, mouse, and primate models of chronic glaucoma show sustained elevation of extracellular ATP and altered purinergic signaling in the posterior eye. Invest Ophthalmol Vis Sci.

[68] Ward MM, Puthussery T, Vessey KA, Fletcher EL (2010). The role of purinergic receptors in retinal function and disease. Adv Exp Med Biol.

[69] Wheeler-Schilling TH, Marquordt K, Kohler K, Guenther E, Jabs R (2001). Identification of purinergic receptors in retinal ganglion cells. Brain Res Mol Brain Res.

[70] Jabs R, Guenther E, Marquordt K, Wheeler-Schilling TH (2000). Evidence for P2X(3), P2X(4), P2X(5), but not for P2X(7) containing purinergic receptors in Muller cells of the rat retina. Brain Res Mol Brain Res.

[71] Monif M, Reid CA, Powell KL, Smart ML, Williams DA (2009). The P2X7 receptor drives microglial activation and proliferation: a trophic role for P2X7R pore. J Neurosci.

[72] Zou J, Vetreno RP, Crews FT (2012). ATP-P2X7 receptor signaling controls basal and TNFalpha-stimulated glial cell proliferation. Glia.

[73] Lister MF, Sharkey J, Sawatzky DA, Hodgkiss JP, Davidson DJ, Rossi AG, Finlayson K (2007). The role of the purinergic P2X7 receptor in inflammation. J Inflamm.

[74] Ridderstrom M, Ohlsson M (2014). Brilliant blue G treatment facilitates regeneration after optic nerve injury in the adult rat. NeuroReport.

[75] Hu H, Lu W, Zhang M, Zhang X, Argall AJ, Patel S, Lee GE, Kim YC, Jacobson KA, Laties AM (2010). Stimulation of the P2X7 receptor kills rat retinal ganglion cells in vivo. Exp Eye Res.

[76] Zhang X, Zhang M, Laties AM, Mitchell CH (2005). Stimulation of P2X7 receptors elevates Ca2+ and kills retinal ganglion cells. Invest Ophthalmol Vis Sci.

[77] Sugiyama T, Lee SY, Horie T, Oku H, Takai S, Tanioka H, Kuriki Y, Kojima S, Ikeda T (2013). P2X7 receptor activation may be involved in neuronal loss in the retinal ganglion cell layer after acute elevation of intraocular pressure in rats. Mol Vis.

[78] Dvoriantchikova G, Ivanov A, Barakat D, Grinberg A, Wen R, Slepak VZ, Shestopalov VI (2012). Genetic ablation of Pannexin1 protects retinal neurons from ischemic injury. PLoS One.

[79] Martin KRG, Quigley HA, Zack DJ, Levkovitch-Verbin H, Kielczewski J, Valenta D, Baumrind L, Pease ME, Klein RL, Hauswirth WW (2003). Gene therapy with brain-derived neurotrophic factor as a protection: retinal ganglion cells in a rat glaucoma model. Invest Ophthalmol Vis Sci.

[80] Hellstrom M, Ruitenberg MJ, Pollett MA, Ehlert EM, Twisk J, Verhaagen J, Harvey AR (2009). Cellular tropism and transduction properties of seven adeno-associated viral vector serotypes in adult retina after intravitreal injection. Gene Ther.

[81] Li Y, Schlamp CL, Nickells RW (1999). Experimental induction of retinal ganglion cell death in adult mice. Invest Ophthalmol Vis Sci.

[82] Bodeutsch N, Siebert H, Dermon C, Thanos S (1999). Unilateral injiury to the adult rat optic nerve causes multiple cellular responses in the contralateral site. J Neurobiol.

[83] Panagis L, Thanos S, Fischer D, Dermon CR (2005). Unilateral optic nerve crush induces bilateral retinal glial cell proliferation. Eur J Neurosci.

[84] Xue LP, Lu J, Cao Q, Hu S, Ding P, Ling EA (2006). Muller glial cells express nestin coupled with glial fibrillary acidic protein in experimentally induced glaucoma in the rat retina. Neurosci.

[85] Lebrun-Julien F, Duplan L, Pernet V, Osswald I, Sapieha P, Bourgeois P, Dickson K, Bowie D, Barker PA, Di Polo A (2009). Excitotoxic death of retinal neurons in vivo occurs via a non-cell-autonomous mechanism. J Neurosci.

[86] Pelzel HR, Schlamp CL, Nickells RW (2010). Histone H4 deacetylation plays a critical role in early gene silencing during neuronal apoptosis. BMC Neurosci.

[87] Pelzel HR, Schlamp CL, Waclawski M, Shaw MK, Nickells RW (2012). Silencing of Fem1c^R3^ gene expression in the DBA/2J mouse precedes retinal ganglion cell death and is associated with Histone Deacetylase activity. Invest Ophthalmol Vis Sci.

[88] Schlamp CL, Johnson EC, Li Y, Morrison JC, Nickells RW (2001). Changes in Thy1 gene expression associated with damaged retinal ganglion cells. Mol Vis.

[89] Dvoriantchikova G, Ivanov A, Panchin Y, Shestopalov VI (2006). Expression of pannexin family of proteins in the retina. FEBS Lett.

[90] Sandilos JK, Chiu YH, Chekeni FB, Armstrong AJ, Walk SF, Ravichandran KS, Bayliss DA (2012). Pannexin 1, an ATP release channel, is activated by caspase cleavage of its pore-associated C-terminal autoinhibitory region. J Biol Chem.

[91] Isenmann S, Wahl C, Krajewski S, Reed JC, Bähr M (1997). Up-regulation of Bax protein in degenerating retinal ganglion cells precedes apoptotic cell death after optic nerve lesion in the rat. Eur J Neurosci.

[92] Nickells RW (2012). The cell and molecular biology of glaucoma: mechanisms of retinal ganglion cell death. Invest Ophthalmol Vis Sci.

[93] Surprenant A, Rassendren F, Kawashima E, North RA, Buell G (1996). The cytolytic P2Z receptor for extracellular ATP identified as a P2X receptor (P2X7). Science.

[94] Franke H, Klimke K, Brinckmann U, Grosche J, Francke M, Sperlagh B, Reichenbach A, Liebert UG, Illes P (2005). P2X7 receptor mRNA and protein in the mouse retina; changes during retinal degeneration in BALBCrds mice. Neurochem Int.

[95] Ishii K, Kaneda M, Li H, Rockland KS, Hashikawa T (2003). Neuron-specific distribution of P2X7 purinergic receptors in the monkey retina. J Comp Neurol.

[96] Vitanova LA, Kupenova PN (2014). Ionotropic purinergic receptors P2X in frog and turtle retina: glial and neuronal localization. Acta Histochem.

[97] Bianchi BR, Lynch KJ, Touma E, Niforatos W, Burgard EC, Alexander KM, Park HS, Yu HP, Metzger R, Kowaluk E (1999). Pharmacological characterization of recombinant human and rat P2X receptor subtypes. Eur J Pharmacol.

[98] Jacobson KA (2010). P2X and P2Y receptors. Tocris Rev.

[99] Boyer JL, Cooper CL, Harden TK (1990). [32P]3′-O-(4-benzoyl)benzoyl ATP as a photoaffinity label for a phospholipase C-coupled P2Y-purinergic receptor. J Biol Chem.

[100] Trueblood KE, Mohr S, Dubyak GR (2011). Purinergic regulation of high-glucose-induced caspase-1 activation in the rat retinal Müller cell line rMC-1. Am J Physiol Cell Physiol.

[101] Franke H, Verkhratsky A, Burnstock G, Illes P (2012). Pathophysiology of astroglial purinergic signalling. Purinergic Signal.

[102] King BF, Townsend-Nicholson A (2008). Involvement of P2Y1 and P2Y11 purinoceptors in parasympathetic inhibition of colonic smooth muscle. J Pharmacol Exp Ther.

[103] Murgia M, Hanau S, Pizzo P, Rippa M, Di Virgilio F (1993). Oxidized ATP, an irreversible inhibitor of the macrophage purinergic P2Z receptor. J Biol Chem.

[104] Stokes L, Spencer SJ, Jenkins TA (2015). Understanding the role of P2X7 in affective disorders—are glial cells the major players?. Front Cell Neurosci.

[105] Morigiwa K, Quan M, Murakima M, Yamashita M, Fukuda Y (2000). P2 purinoceptor expression and functional changes of hypoxia-activated cultured rat retinal microglia. Neurosci Lett.

[106] Ahlers KE, Karacay B, Fuller L, Bonthius DJ, Dailey ME (2015). Transient activation of microglia following acute alcohol exposure in developing mouse neocortex is primarily driven by BAX-dependent neurodegeneration. Glia.

[107] Luo C, Yang X, Kain AD, Powell DW, Kuehn MH, Tezel G (2010). Glaucomatous tissue stress and the regulation of immune response through glial toll-like receptor signaling. Invest Ophthalmol Vis Sci.

[108] Nakamura J, Meguro A, Ota M, Nomura E, Nishide T, Kashiwagi K, Mabuchi F, Iijima H, Kawase K, Yamamoto T (2009). Association of toll-like receptor 2 gene polymorphisms with normal tension glaucoma. Mol Vis.

[109] Shibuya E, Meguro A, Ota M, Kashiwagi K, Mabuchi F, Iijima H, Kawase K, Yamamoto T, Nakamura M, Negi A (2008). Association of Toll-like receptor 4 gene polymorphisms with normal tension glaucoma. Invest Ophthalmol Vis Sci.

[110] Morzaev D, Nicholson JD, Caspi T, Weiss S, Hochhauser E, Goldenberg-Cohen N (2015). Toll-like receptor-4 knockout mice are more resistant to optic nerve crush damage than wild-type mice. Clin Exp Ophthalmol.

[111] Dvoriantchikova G, Barakat D, Hernandez E, Shestopalov VI, Ivanov D (2010). Toll-like receptor 4 contributes to retinal ischemia/reperfusion injury. Mol Vis.

[112] Goulopoulou S, Matsumoto T, Bomfim GF, Webb RC (2012). Toll-like receptor 9 activation: a novel mechanism linking placenta-derived mitochondrial DNA and vascular dysfunction in pre-eclampsia. Clin Sci.

[113] Chinnery PF, McLenachan S, Binz N, Sun Y, Forrester JV, Degli-Esposti MA, Pearlman E, McMenamin PG (2012). TLR9 lingand CpG-OND appliced to the injured mouse cornea elicits retinal inflammation. Am J Pathol.

[114] Locovei S, Scemes E, Qiu F, Spray DC, Dahl G (2007). Pannexin1 is part of the pore forming unit of the P2X7 receptor death complex. FEBS Lett.

[115] Matsumoto K, Anzai M, Nakagata N, Takahashi A, Takahashi Y, Miyata K (1994). Onset of paternal gene activation in early mouse embryos fertilized with transgenic mouse sperm. Mol Reprod Dev.

[116] Penuela S, Gehi R, Laird DW (1828). The biochemistry and function of pannexin channels. Biochim Biophys Acta.

[117] Deckwerth TL, Elliot JL, Knudson CM, Johnson EM, Snider WD, Korsmeyer SJ (1996). BAX is required for neuronal death after trophic factor deprivation and during development. Neuron.

[118] White FA, Keller-Peck CR, Knudson CM, Korsmeyer SJ, Snider WD (1998). Widespread elimination of naturally occurring neuronal death in bax-deficient mice. J Neurosci.

[119] Sun W, Oppenheim RW (2003). Response of motoneurons to neonatal sciatic nerve axotomy in Bax-knockout mice. Mol Cell Neurosci.

[120] Uo T, Kinoshita Y, Morrison RS (2005). Neurons exclusively express N-Bak, a BH3 domain-only Bak isoform that promotes neuronal apoptosis. J Biol Chem.

[121] Kawai K, Itoh T, Itoh A, Horiuchi M, Wakayama K, Bannerman P, Garbern JY, Pleasure D, Lindsten T (2009). Maintenance of the relative proportion of oligodendrocytes to axons even in the absence of BAX and BAK. Eur J Neurosci.

[122] Li Y, Semaan SJ, Schlamp CL, Nickells RW (2007). Dominant inheritance of retinal ganglion cell resistance to optic nerve crush in mice. BMC Neurosci.

[123] Mosinger Ogilvie J, Deckwerth TL, Knudson CM, Korsmeyer SJ (1998). Suppression of developmental retinal cell death but not photoreceptor degeneration in Bax-deficient mice. Invest Ophthalmol Vis Sci.

[124] Hanisch UK (2013). Functional diversity of microglia—how heterogeneous are they to being with?. Front Cell Neurosci.

