# Supplementary figures and images for: Retinal glial responses to optic nerve crush are attenuated in Bax-deficient mice and modulated by purinergic signaling pathways
Source: J Neuroinflammation. 2016 Apr 28;13:93. doi: 10.1186/s12974-016-0558-y (PMC4850653; doi:10.1186/s12974-016-0558-y)

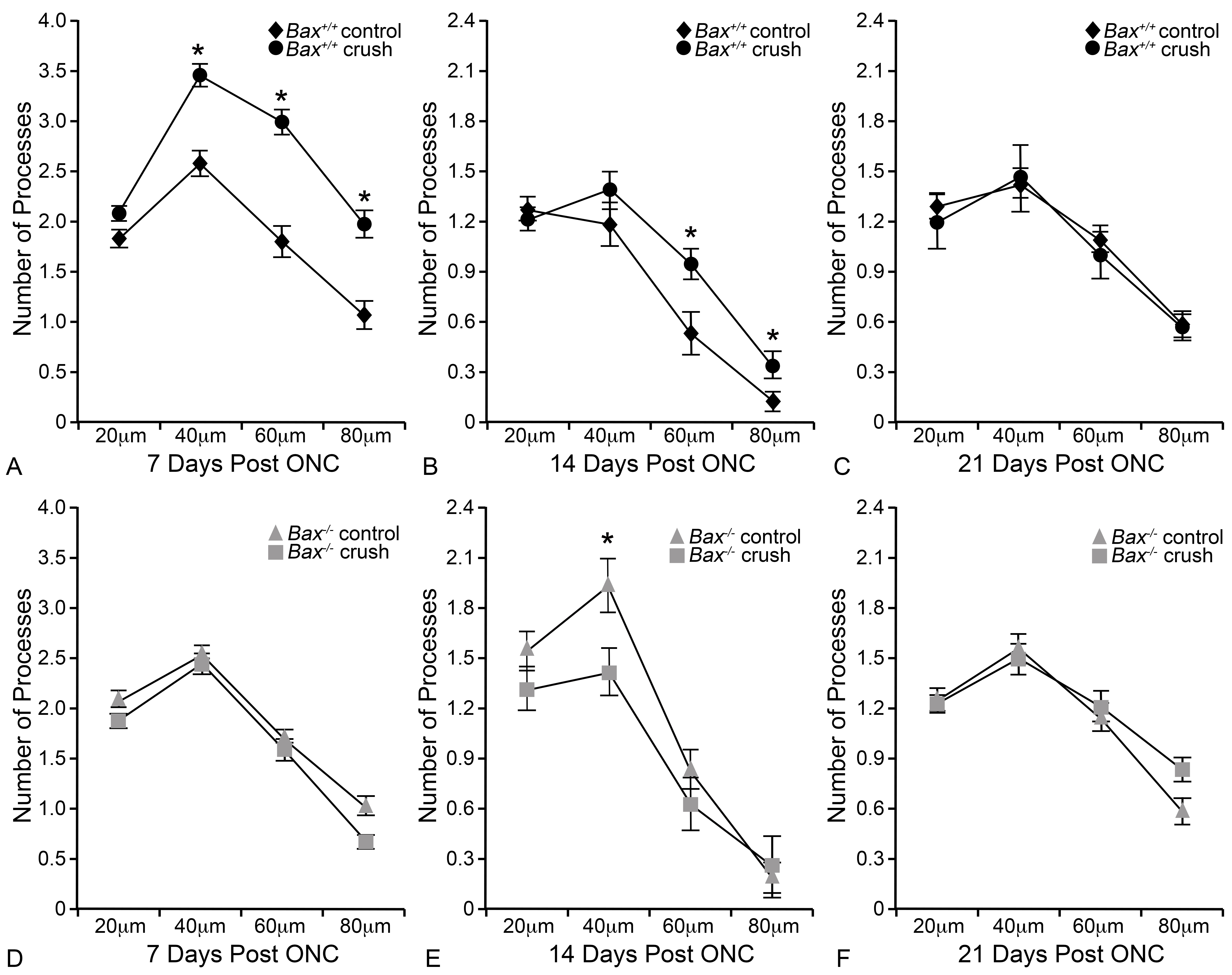

Supplement: Additional file 1: Figure S1. — Summary of Sholl analysis of AIF1-expressing microglia after optic nerve crush. (A–C) Wild-type retinas at 7, 14, and 21 days after optic nerve crush, respectively. (D–F) Bax−/− retinas at 7, 14, and 21 days, respectively. Data is presented as mean ± SE. *P < 0.05. Each graph represents a minimum of 50 cells measured from three retinas of each genotype. (JPG 2.19 mb) [file 12974_2016_558_MOESM1_ESM.jpg]
